# Vascular remodeling by placenta-derived mesenchymal stem cells restores ovarian function in ovariectomized rat model via the VEGF pathway

**DOI:** 10.1038/s41374-020-00513-1

**Published:** 2020-12-10

**Authors:** Jinki Cho, Tae-Hee Kim, Jin Seok, Ji Hye Jun, Hyeri Park, Minyeoung Kweon, Ja-Yun Lim, Gi Jin Kim

**Affiliations:** 1 Department of Biomedical Science, CHA University, Seongnam, Gyeonggi-Do 13488 Republic of Korea; 2 Department of Obstetrics and Gynecology, Soonchunhyang University College of Medicine Hospital, Bucheon, Gyunggi-do 14584 Republic of Korea; 3 College of Life Science, University of Glasgow, Glasgow, Scotland G12 8QQ UK; 4 Department of Health and Environmental Science, Korea University, Seoul, 02481 Republic of Korea

**Keywords:** Mesenchymal stem cells, Infertility

## Abstract

Angiogenesis plays an important role in damaged organ or tissue and cell regeneration and ovarian development and function. Primary ovarian insufficiency (POI) is a prevalent pathology in women under 40. Conventional treatment for POI involves hormone therapy. However, due to its side effects, an alternative approach is desirable. Human mesenchymal stem cells (MSCs) from various sources restore ovarian function; however, they have many limitations as stem cell sources. Therefore, it is desirable to study the efficacy of placenta-derived MSCs (PD-MSCs), which possess many advantages over other MSCs, in a rat model of ovarian dysfunction. Here, we investigated the restorative effect of PD-MSCs on injured ovaries in ovariectomized (OVX) rats and the ability of intravenous transplantation (Tx) of PD-MSCs (5 × 10^5^) to enhance ovarian vasculature and follicular development. ELISA analysis of serum revealed that compared to the non-transplantation (NTx) group, the Tx group showed significantly increased levels of anti-Müllerian hormone, follicle stimulating hormone, and estradiol (E2) (*P < 0.05). In addition, histological analysis showed more mature follicles and less atresia and restoration of expanded blood vessels in the ovaries of the OVX PD-MSC Tx group than those of the NTx group (*P < 0.05). Furthermore, folliculogenesis-related gene expression was also significantly increased in the PD-MSC Tx group (*P < 0.05). Vascular endothelial growth factor (VEGF) and VEGF receptor 2 expressions were increased in the ovaries of the OVX PD-MSC Tx group compared to the NTx group through PI3K/AKT/mTOR and GSK3β/β-catenin pathway activation. Interestingly, ex vivo cocultivation of damaged ovaries and PD-MSCs or treatment with recombinant VEGF (50 ng/ml) increased folliculogenic factors and VEGF signaling pathways. Notably, compared to recombinant VEGF, PD-MSCs significantly increased folliculogenesis and angiogenesis (*P < 0.05). These findings suggest that VEGF secreted by PD-MSCs promotes follicular development and ovarian function after OVX through vascular remodeling. Therefore, these results provide fundamental data for understanding the therapeutic effects and mechanism of stem cell therapy based on PD-MSCs and provide a theoretical foundation for their application for obstetrical and gynecological diseases, including infertility and menopause.

## Abstract (web summary)

Vascular endothelial growth factor secreted by placenta-derived mesenchymal stem cells (PD-MSCs) promotes follicular development and ovarian function after ovariectomy through vascular remodeling. These results provide fundamental data for understanding the therapeutic mechanisms of stem cell therapy based on placenta-derived mesenchymal stem cells PD-MSCs and provide a theoretical foundation for their application for obstetrical and gynecological diseases, including infertility and menopause.

## Introduction

The ovary is a prominent organ that regulates the balance of reproductive hormones and the periodical development of mature oocytes during the menstrual cycle. Primary ovarian insufficiency (POI), also called premature ovarian failure (POF), is a prevalent degenerative disease of the ovary in women under the age of 40 years and is an important cause of infertility [1–4]. The prevalence of POI has been estimated to be 0.5% in women under 35 years of age and 1% in women under 40 years of age [2, 5]. Although many studies have reported the etiological factors that cause various types of ovarian dysfunction, including genetic alterations, toxins, and autoimmunity, which contribute to the disruption of folliculogenesis, the follicle maturation process, the exact mechanisms of POI are not fully understood [6–8]. POI is characterized by amenorrhea, increased follicle stimulating hormone (FSH) levels, decreased estrogen (E2) levels, and menstrual irregularities [6]. Dysregulated gonadotropin and steroid hormones affect the process of folliculogenesis and the follicle reserve, leading to a diminished follicular pool [9]. Current treatment for POI mainly focuses on hormone replacement therapy (HRT); however, HRT alleviates only the symptoms but not the disease, and does not restore ovarian reserve [10, 11]. Therefore, it is urged to seek more effective treatment strategies.

For the normal ovarian development, systematized new blood vessel formation and regression are precisely controlled by hormonal levels. As an ovarian follicle develops, establishment of the vasculature provides access to nutrients and hormones [12]. The continued development of vasculature complexity along with follicular growth indicates a close relationship between these processes, known as follicular angiogenesis [13]. Among the many proangiogenic factors, vascular endothelial growth factor (VEGF) is one of the major candidates for the regulation of angiogenesis in the ovary [14]. Therefore, insufficient vascular supply could limit growth and lead to atresia [13]. It has been reported that abnormal angiogenesis is involved in the progression of degenerative ovarian diseases, such as polycystic ovary syndrome (PCOS) and ovarian cancer [15, 16].

Recently, stem cell therapy using mesenchymal stem cells (MSCs), which are multipotent adult stem cells with the potential to differentiate into cells of the three germ layers, was shown to improve ovarian dysfunction [17–19]. Wang et al. reported that human umbilical cord MSCs increase follicular development by reducing apoptosis in a mouse model of POF [20]. Moreover, human bone marrow mesenchymal stromal cells also reactivate folliculogenesis and restore ovarian hormone production [21]. Although transplanted MSCs improve ovarian function, including by changing hormone levels in the blood and altering folliculogenesis-related genes, further study on how transplanted stem cells regulate the therapeutic mechanism and which factors are involved is needed.

Placenta-derived mesenchymal stem cells (PD-MSCs), which have recently attracted much attention, are derived from the human placenta and are known to promote angiogenesis and have therapeutic effects in liver regeneration. PD-MSCs have many advantages, such as high multipotency and immunomodulatory ability, the ability to be obtained from young donors and in large quantities, and the avoidance of ethical issues associated with embryonic stem cells [22–25]. These advantages make PD-MSCs great candidates for regenerative medicine and allow their use in many studies on stem cell therapy [26]. In our previous study, we showed that PD-MSCs have strong therapeutic effects by applying spheroid PD-MSCs in ovariectomized rat model [27]. After being transplanted in OVX rats, PD-MSC spheroids induce folliculogenesis in vivo through enhanced engraftment efficacy; however, the underlying mechanism still needs to be elucidated. Therefore, the objectives of the study were to analyze the effect of transplanted PD-MSCs on the ovarian vasculature in ovarian tissues of OVX rats and investigate the correlation between the ovarian vasculature and folliculogenesis in ovarian functions. Finally, we determined which factors of PD-MSCs are involved in improving the ovarian vasculature and follicular development and the regulatory mechanism.

## Materials and methods

### Cell culture

Human PD-MSCs were isolated from the chorionic plate of the placenta with approval by the Institutional Review Board of CHA General Hospital, Seoul, Republic of Korea (IRB 08-17). PD-MSCs were obtained from the human placentas of the consented women after uncomplicated elective Caesarean delivery by separating via blunt dissection from the placental body. Then the samples were characterized by examining their morphology and expression of stem cell markers, along with FACS analysis of the surface markers. The details of the method and results can be found in our previous studies [23, 24]. PD-MSCs were cultured in alpha-minimum essential medium (α-MEM, HyClone Laboratories Inc., South Logan, UT, USA) with 10% fetal bovine serum (FBS, GIBCO-BRL, Langley, OK, USA), 1% penicillin/streptomycin (GIBCO-BRL), 25 ng/mL FGF-4 (Peprotech, Rocky Hill, NJ, USA), and 1 μg/mL heparin (Sigma-Aldrich, St. Louis, MO, USA) at 37 °C in a humidified 5% CO_2_/95% air atmosphere, and stained with PKH67 by using a PKH67 Fluorescent Cell Linker Kit (Sigma-Aldrich).

### Animal model and transplantation of PD-MSCs

Seven-week-old female Sprague-Dawley rats (Orient Bio Inc., Seongnam, Republic of Korea) were maintained in an air-conditioned animal facility. Ovariectomy was performed by surgically removing one of the ovaries under general anesthesia with avertin (2,2,2-tribromoethanol, Sigma-Aldrich). Each rat was anesthetized by abdominal injection of avertin, and the operation was performed after the animals were fully anesthetized. A total of 70 rats were used: 60 rats for in vivo experiments and 10 for ex vivo experiments. There were 5 rats in each of the following groups: the normal group, non-transplantation (NTx) group, and transplantation (Tx) group. The animals were randomly assigned to one of the groups. One week after OVX, PD-MSCs (5 × 10^5^ cells, 8–10 passages) were injected intravenously into the Tx group through the tail vein. The rats were sacrificed after 1, 2, 3, or 5 weeks, and ovarian tissues and blood samples were collected. The blood sample of each individual was centrifuged at 1300 rcf for 15 min, and then serum was collected for hormone analyses, namely FSH, E2, and AMH. The experimental procedures for animal model establishment and the experiments were approved by the Institutional Animal Care and Use Committee of CHA University, Seongnam, Republic of Korea (IACUC-140009).

### Quantitative real-time polymerase chain reaction (qRT-PCR) analysis

The ovarian tissues of the rats were harvested and cryofrozen in liquid nitrogen. The tissues were then homogenized and lysed using liquid nitrogen, and total RNA was isolated. Each sample was mixed with 1 ml of TRIzol (Invitrogen, Carlsbad, CA, USA) and 0.2 ml of chloroform and then centrifuged to collect the supernatant. The isolated supernatant was washed with isopropyl alcohol/ethyl alcohol and discarded, and the pellet was collected as the final product. The pellet was then dissolved in DEPC-treated water (Invitrogen). Total RNA (1,000 ng) was used to synthesize cDNA using Superscript III reverse transcriptase (Invitrogen). The PCR conditions for cDNA synthesis were as follows: 5 min at 65 °C, 1 min at 4 °C, 60 min at 50 °C, and 15 min at 72 °C. cDNA was used for qRT-PCR analysis, which was performed with SYBR Ex taq (Roche, Basel, Switzerland). The cDNA was subsequently amplified by PCR under the following conditions: 5 s at 95°C and 40 cycles of 95 °C for 5 s and 60 °C for 30 s. The sequences of the qRT-PCR primers are listed in Table 1. *rGapdh* was used as an internal control for normalization, and each sample was analyzed in triplicate.

**Table 1:** Primer sequences using quantitative real time polymerase chain reaction.

Gene	Forward primer	Reverse primer	Tm (°C)
hAlu	5′-GGA GGC TGA GGC AGG AGA A-3′	5′-CGG AGT CTC GCT CTG TCG CCC A-3′	58
Lhx8	5′-GTATCACTTGGCTTGCTT-3′	5′-ATTACCGTTCTCCACTTC-3′	55
Nanos3	5′-CTCTGCATGAGGAAGAGGAGCC-3′	5′-GGACTGATAGATCGCACGAGA-3′	55
Lin28a	5′-CCCGGTGGACGTCTTTGTG-3′	5′-CACTGCCTCACCCTCCTTGA-3′	58
Nobox	5′-AGCCAGTGCAGATCTGCACC-3′	5′-TGTCACTGCCAGGAACATCCCTC-3′	50
BMP15	5′-ATCTGATGTCCCTTGTCCTT-3′	5′-CTCTGTATTGATGGCATGGTT-3′	54
EGFR	5′-AGATTGCAAAGGGCATGAACTAC-3′	5′-ACATTCCTGGCTGCCAAGCT-3′	58
VEGF	5′-ACT GGA CCC TGG CTT TAC TG-3′	5′-ACG CAC TCC AGG GCT TCA TC-3′	56
VEGFR1	5′-CCA CAC CTG AAA TCT ACC AA-3′	5′-TGG GGACT GAG TAT GTG AAG-3′	55
VEGFR2	5′-AAGCAAATGCTCAGCAGGAT-3′	5′-TAG GCA GGG AGA GTC CAG AA-3′	58
Endoglin	5′-AAG GTG TGA CTG TAC ACA AG-3′	5′-CCA GAT CTG CAT ATT GTG GT-3′	55

### Western blot analysis

Homogenized cells and ovarian tissues were lysed with RIPA buffer containing protease inhibitor cocktail (Roche) and phosphatase inhibitor (Sigma-Aldrich) on ice. After centrifugation, the supernatant was collected. The protein lysates were quantified using a bovine serum albumin (BSA) protein assay kit (Sigma-Aldrich) and a microplate reader (Molecular Devices, San Jose, CA, USA). Based on the protein concentrations, 20 μg of each protein extracts was measured and used for 8–15% sodium dodecyl sulfate polyacrylamide gel electrophoresis. The separated proteins were transferred to polyvinylidene difluoride membranes (Bio-Rad Laboratories, Hercules, CA, USA) and blocked in blocking buffer containing 0.1% Tween 20 and 5% BSA in Tris-buffered saline (TBS) at room temperature for 1 h. The membranes were incubated with antibodies at 4 °C for 16 h. The antibodies used in this study were as follows: α-Tubulin (CP06-100UG, Sigma-Aldrich), GAPDH (LF-PA0018, AbFrontier, Seoul, Republic of Korea), nonphospho-β-catenin (4270 S, Cell Signaling Technology, Danvers, MA, USA), phospho-protein kinase B (AKT) (9271 S, Cell Signaling), AKT (9272 S, Cell Signaling), VEGF (NB100-664, Novus Biologicals, Centennial, CO, USA), VERGFR2 (9698 S, Cell Signaling), LIN28 (AB63740, Abcam, Cambridge, UK), NANOS3 (AB70001, Abcam), LHX8 (SC-22217, Santa Cruz Biotechnology, Dallas, TX, USA), CD31 (SC-1506, Santa Cruz Biotechnology), NOBOX (SC-390016, Santa Cruz Biotechnology), EGFR (2232, Cell Signaling), BMP15 (MBS2516631, MyBioSource, San Diego, CA, USA), phospho-GSK3α/β (9331 S, Cell Signaling), GSK3α/β (5676, Cell Signaling), phospho-mTOR (AB109268, Abcam), mTOR (2983 S, Cell Signaling), and phosphatidylinositol-3-kinase (PI3K(p110α)) (4255 S, Cell Signaling). After incubation, the membranes were washed with TBS and treated with a horseradish peroxidase-conjugated secondary antibody according to the corresponding host at room temperature for 1 h. The membranes were treated with a Clarity Western ECL kit (Bio-Rad Laboratories) and the protein bands detected with a ChemiDoc XRS + imaging system (Bio-Rad Laboratories). The blots were also analyzed by ImageJ software (NIH, Bethesda, MD, USA) for detailed comparisons.

### Histological analysis

Ovarian tissues were fixed in 10% formalin and embedded in paraffin. The samples were sectioned at a thickness of 5 μm. Two of every seven serial sections were used for hematoxylin and eosin (H&E) staining. The sectioned samples were stained with H&E and observed under a microscope at ×10, ×100, ×200, or ×400 magnification. Follicle counts and ovarian blood vessel measurements were performed with Caseviewer software (3DHistech, Budapest, Hungary). All follicles within the selected ovarian sections were counted and classified by follicle stage. The stages consisted of the primordial, primary, secondary and antral stages, which are part of normal ovarian development, and follicles in these stages were counted to determine the total number of follicles However, atretic follicles were disregarded from the total count. All experiments were conducted in duplicate.

### Immunofluorescence staining

To investigate the localization of vascular epithelial growth factor receptor 2 (VEGFR2), β-catenin, and CD31 in ovarian tissues, 6-μm-thick frozen ovarian sections were treated with Blocking Solution (DAKO, Glostrup, Productionsvej, Denmark) at room temperature for 60 min and treated with primary antibodies against each target protein at 4 °C overnight. The following antibodies were used: rabbit anti-nonphospho-β-catenin (4270 S, Cell Signaling) diluted 1:100; rabbit anti-VEGFR2 (9698 S, Cell Signaling) diluted 1:100; and goat anti-CD31 (SC-1506, Stan Cruz Biotechnology) diluted 1:100. On the following day, the tissues were washed with phosphate-buffered saline (PBS) and incubated with an Alexa 488-conjugated secondary antibody or an Alexa 568-conjugated secondary antibody (Invitrogen) at room temperature for 60 min. Then, the sections were stained with 4,6-diamidino-2-phenylindole (DAPI) for nuclear counterstaining. The slides were observed by fluorescence microscopy (Nikon, Tokyo, Minato, Japan). All parts of each slide were observed, and representative images were captured for presentation.

### Indirect cocultivation system and treatment with recombinant VEGF

An indirect cocultivation system was established with a 24-well insert system (BD Biosciences, Franklin, NJ, USA). Each ovary harvested from the rats was cut in half and cocultivated with PD-MSCs. Matrigel was solidified in a 24-well plate in which the ovaries were to be embedded. Extracellular fat reserves were then removed from the ovaries, and the ovaries were rinsed with Dulbecco’s phosphate-buffered saline (DPBS, Welgene, Gyeongsan-Si, Republic of Korea) and α-MEM (HyClone Laboratories Inc.) containing 10% FBS (GIBCO-BRL) and 1% penicillin/streptomycin (GIBCO-BRL). The collected and cut ovaries were each embedded in a 24-well plate, and 1 × 10^4^ PD-MSCs were seeded on an insert that was then placed over the 24-well plate containing the ovary and medium. The cocultivated ovaries were then collected after 24 or 48 h for analysis. To compare the effect of VEGF treatment and PD-MSC cocultivation, the cut ovaries were placed on Matrigel in 24-well plates and treated with recombinant human VEGF 165 (50 ng/ml, CreaGene, Seongnam, Republic of Korea) in the culture medium. The samples were collected after 24 or 48 h for analysis.

### Statistical analysis

All experiments were conducted in duplicate or triplicate. The results are presented as the mean ± standard error. Student’s *t* tests were used to analyze group-wise comparisons, a *P* value < 0.05 indicated statistical significance.

## Results

### Engraftment of PD-MSCs in OVX rats

**Fig. 1:**
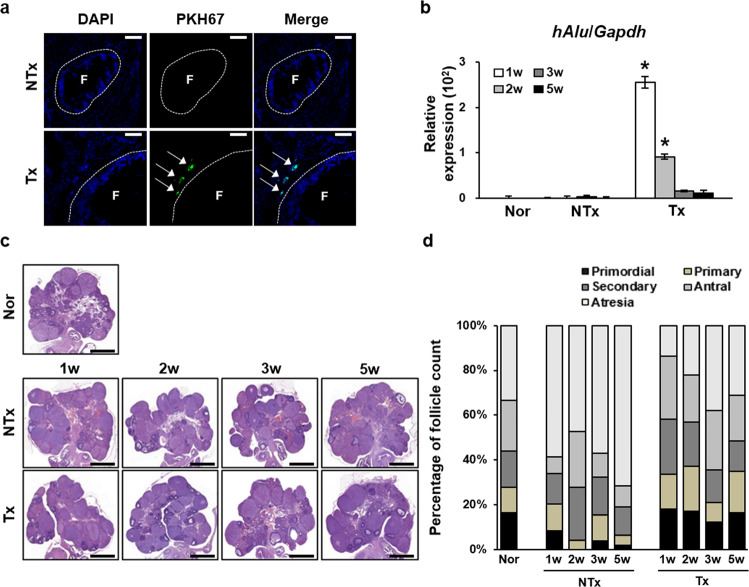
Homing of PD-MSCs to the ovary and ovarian follicle counts. **a** Immunofluorescence staining confirmed that PKH67 (green) was expressed near follicles within the ovarian tissues of the PD-MSC group but was not expressed within those of the NTx group. Nuclei were stained with DAPI (blue). Arrow, PKH67 fluorescence. F, ovarian follicle. Scale bar: 25 μm. **b** The mRNA expression level of *hAlu* in ovarian tissue samples confirmed the successful localization and expression of PD-MSCs at the ovaries in the Tx group, whereas the opposite was observed in the normal and NTx groups. *vs. the normal group (P < 0.05). **c** H&E staining of ovarian tissues. **d** The graph of the percentage of follicles in different stages of folliculogenesis indicates an increased number of total follicles in all stages of development in the Tx group compared to the NTx group. The ovarian tissues were collected 2 weeks after the transplantation. Nor, normal group. NTx, non-transplantation group. Tx, transplantation group. 1w, week 1. 2w, week 2. 3w, week 3. 5w, week 5.

To investigate the therapeutic effects of human PD-MSCs on a model of ovarian dysfunction, rats underwent ovariectomy according to the protocol described in the methods section. PD-MSCs were transplanted into OVX rats via tail vein injection after ovariectomy. To confirm the engrafting ability of the cells in vivo, PD-MSCs were labeled with PKH67, and confocal microscopy was used to detect the fluorescent signal in the ovarian tissues of the 2-week Tx group (Fig. 1a). PKH67 fluorescence was detected near follicles in ovarian tissues; however, fluorescence was not observed in the NTx group. In addition, mRNA expression of the human-specific *Alu* sequence (*hAlu*) was analyzed to quantitatively measure engrafted human cells. Expression of *hAlu* was high in the Tx group from the first week until the second after Tx and then gradually decreased over time (Fig. 1b, *P* < 0.05). In contrast, the NTx group did not show significant expression of *hAlu*. Taken together, the *hAlu* expression level and PKH67 fluorescence data suggest that PD-MSCs became engrafted in the ovary in vivo, even though they were transplanted via tail vein injection.

### PD-MSC Tx increased the number of mature follicles and decreased atrophy

To examine the folliculogenic effect of PD-MSCs, ovarian sections from the different groups were analyzed by H&E staining (Fig. 1c). Histological analysis was conducted to observe the structure of the follicles within the ovarian tissues. The ovaries of the control group exhibited normal folliculogenesis, with the numbers of follicles spread evenly throughout the different stages of development (Fig. 1d). In contrast, compared to the normal group, the NTx group showed a significant degree of atrophy and a decreased number of follicles in normal developmental stages, especially the antral stage (*P < 0.05). The increase in the degree of atresia was significantly greater in the NTx group than the Tx group (*P < 0.05). The number of growing follicles was significantly decreased in the NTx group compared to the normal group (*P < 0.05). However, compared to the NTx group, the PD-MSC Tx group displayed a significant increase in the number of follicles in all stages as well as a decrease in the degree of atrophy (*P < 0.05). The numbers of antral follicles in the PD-MSC Tx groups were significantly greater than those in the respective NTx groups (*P < 0.05). These data indicate that engrafted PD-MSCs triggered follicular development as well as a decrease in the degree of atrophy in the OVX model.

### PD-MSC Tx promoted the upregulation of folliculogenesis-related genes

**Fig. 2:**
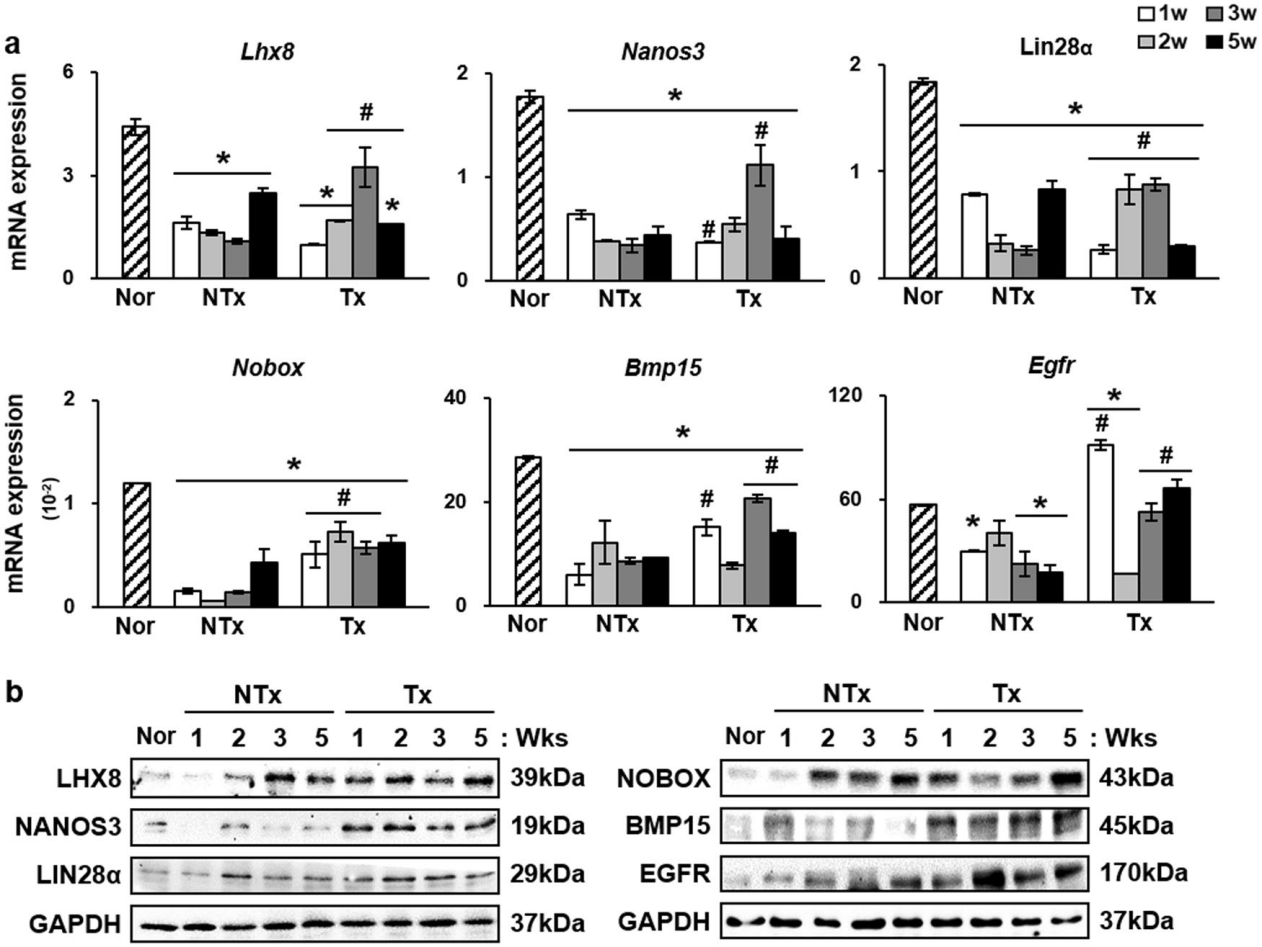
Upregulation of folliculogenic factors at the mRNA and protein levels after PD-MSC transplantation. **a** mRNA expression levels of *Nanos3, Lhx8, Lin28α, Nobox, Bmp15, and Egfr* were decreased in the NTx groups compared to the normal group and increased in the Tx groups compared to the NTx groups with some exceptions. **b** Western blot analysis showed an overall increase in the expression level in the Tx groups compared to the NTx groups with some exceptions. **c** Immunohistochemistry for LHX8 showed higher folliculogenic activity after Tx. PD-MSCs, treatment group. *vs. the normal group (*P* < 0.05). #, vs. NTx (*P* < 0.05). Nor, normal group. NTx, non-transplantation group. Tx, transplantation group. 1w, week 1. 2w, week 2. 3w, week 3. 5w, week 5.

Generally, enhanced follicular development is controlled by the expression of genes known to be upregulated within growing follicles, such as *Lhx8, Nanos3, Lin28α, Nobox, Bmp15*, and *Egfr* [7]. Therefore, we evaluated the mRNA and protein expression levels of these factors by qRT-PCR and western blot analyses. Compared to those in the normal group, the overall mRNA expression levels of *Lhx8, Nanos3, Lin28α, Nobox, Bmp15*, and *Egfr* were significantly downregulated in the NTx groups (Fig. 2a). The relative mRNA expression of *Nanos3*, which was normalized to *Gapdh* expression, was higher in the normal group than in the 1-, 2-, 3-, and 5-Tx groups (*P < 0.05). However, an increase in the expression level of *Nanos3* was observed in the 3-week Tx group compared to the respective NTX group (*P < 0.05). In addition, the 2- and 3-week Tx groups also showed higher expression of *Lhx8* and *Lin28α* than the respective NTx groups (*P < 0.05). At the protein level, an increase in the expression of LHX8 was observed in the 1-, 2-, and 5-week Tx groups compared to the respective NTx groups (*P = 0.000, *P = 0.009, and *P = 0.0236, respectively) (Fig. 2b). NANOS3 protein expression levels were increased in the 1- and 5-week Tx groups compared to the respective NTx groups (*P = 0.030 and *P = 0.015, respectively). While not all groups showed the same pattern of change in the expression levels of *Lhx8, Nanos3, Lin28α, Nobox, Bmp15*, and *Egfr*, the expression of folliculogenesis-related genes after PD-MSC Tx was increased (Supplementary Fig. 1). These data show that the development of follicles after PD-MSC Tx is associated with increased expression of folliculogenesis-related genes.

### Serum levels of AMH, FSH, and E2 were upregulated after Tx

In general, the secretion and balance of endocrinal hormones are closely involved in the development of follicles in ovarian tissue and also regulate the function of the ovary [28]. To confirm the effect of PD-MSCs, we analyzed the change in the levels of reproductive hormone, such as serum anti-Müllerian hormone (AMH), FSH and estradiol (E2), in the blood serum using ELISA (Table 2). AMH is known to induce folliculogenesis during the first phase in which primordial follicles develop into primary follicles [29]. Blood from each sample was harvested and Compared to the NTx group, the Tx group showed a gradual increase in the concentration of serum AMH as time progressed. The serum AMH level did not significantly increase during week 1; however, starting in week 2, the Tx group showed a significant increase in the serum AMH level compared to that in the NTx group (*P < 0.05). The increase in AMH in the Tx groups suggests that folliculogenesis was induced in the primary recruitment and development stages. A significant increase in the serum FSH level was detected in the 2- and 5-week PD-MSC Tx groups compared to the respective NTx groups (*P < 0.05). An increase in FSH may indicate an increase in secondary follicle development because FSH acts as a key mediator of primary follicle development into secondary follicles and as a preserver of follicles [30, 31]. The serum E2 level in the PD-MSC Tx group was significantly higher than that in the NTx group (*P < 0.05). These data are consistent with the involvement of PD-MSCs in ovarian development.

**Table 2:** AMH, FSH, and E2 serum levels in vivo.

Group		Week 1	Week 2	Week 3	Week 5
AMH (pg/ml)	NTx	0.830 ± 0.001	0.870 ± 0.084	1.453 ± 0.004	1.273 ± 0.027
	Tx	0.589 ± 0.004	1.742 ± 0.089^*^	1.603 ± 0.125	2.159 ± 0.098^*^
FSH (IU/L)	NTx	6.344 ± 0.316	4.637 ± 0.109	4.661 ± 0.010	5.593 ± 0.040
	Tx	5.278 ± 0.040^*^	5.157 ± 0.020^*^	4.165 ± 0.099	7.135 ± 0.174^*^
E2 (pg/ml)	NTx	22.541 ± 8.356	51.882 ± 1.924	47.802 ± 5.372	17.748 ± 3.141
	Tx	43.009 ± 8.430^*^	67.428 ± 8.192^*^	40.806 ± 19.786	60.044 ± 9.769^*^

*vs. NTx (*P* < 0.05).

### PD-MSCs restored the structure of blood vessels in injured ovarian tissues

**Fig. 3:**
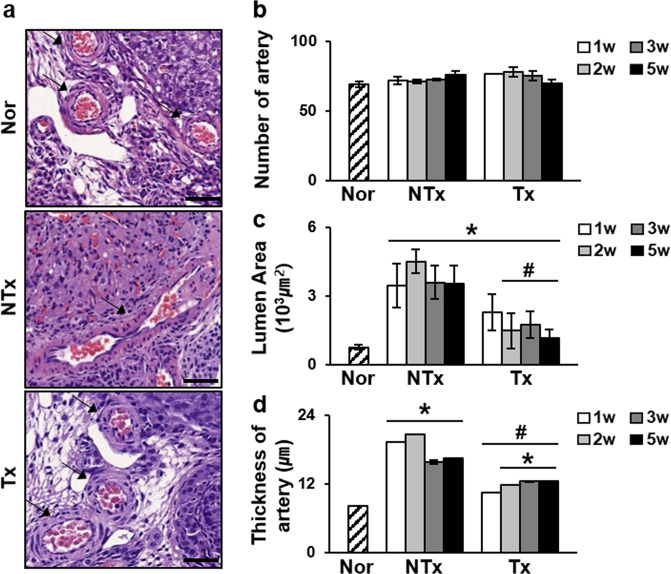
Measurements of the blood vessel luminal area and wall thickness. **a** H&E staining of ovarian sections. Arrow, blood vessel. Scale bar: 1.5 mm. **b** The number of blood vessels within the ovaries of different groups appeared to be similar. **c** The luminal area of the blood vessels was increased in the NTx group compared to the normal group but was restored in the Tx group. **d** The same pattern was also observed for wall thickness. However, the number of blood vessels within the ovarian tissues remained relatively constant. *vs. the normal group (*P* < 0.05). # vs. the NTx group (P < 0.05). Nor normal group, NTx non-transplantation group, Tx transplantation group.

The development of follicles in ovarian tissues parallels the development of blood vessels, and abnormal development of blood vessels reduces ovarian function [32]. As shown in Fig. 3, the pattern of blood vessel vascular structures within the ovaries was different in the NTx group compared to the normal and Tx groups (Fig. 3a). In the NTx group, blood vessel exhibited an irregular and elongated form, whereas the blood vessels in the Tx group exhibited a structure similar as those in the normal group. Therefore, we measured the numbers of vessels, the thickness of the wall and the luminal area of the blood vessels and compared data between the groups. Although the overall numbers of blood vessels in the ovaries of the different groups did not vary significantly, the blood vessels of the NTx group were in elliptical structure with thicker walls and wider luminal areas than the blood vessels of the normal group, which maintained a relatively circular shape. The luminal area and wall thickness of the normal group were 770.6 ± 113.2 µm^2^ and 8.25 ± 0.03 µm, respectively. However, in the NTx group, the luminal area was significantly expanded (*P < 0.05) (Fig. 3c, d). Notably, the structural change observed in the NTx groups compared to the normal group were recovered in the Tx group, with the thickness of the wall as well as luminal area being reduced (*P < 0.05). These data may suggest that PD-MSCs restored abnormally expanded and thickened blood vessel structures without increasing the number of blood vessels.

### PD-MSCs induced the expression of VEGF and VEGFR2 and activation of the VEGF signaling pathway

**Fig. 4:**
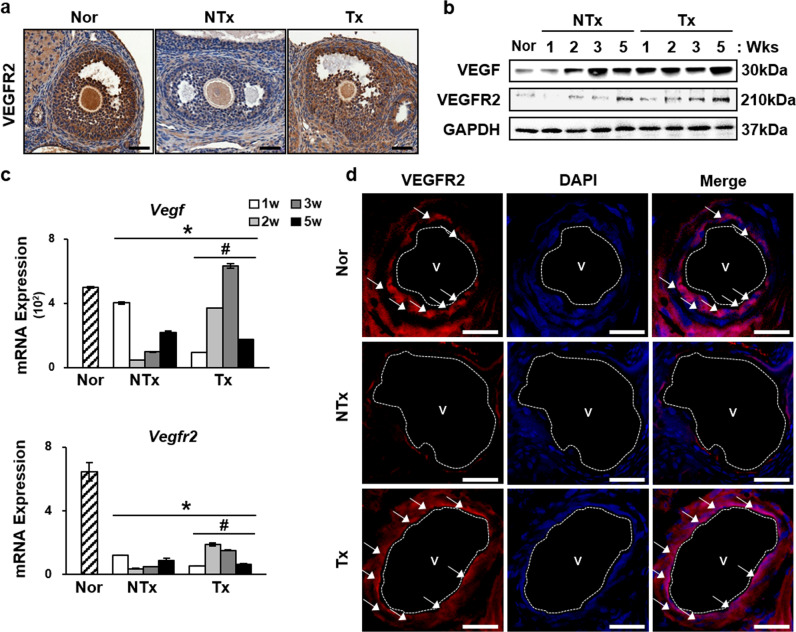
Increased mRNA and protein expression levels of *Vegf* and *Vegfr2*. **a** Immunohistochemistry staining showed high expression of VEGFR2 in antral follicles of Tx group compared to the NTx group. **b** Protein levels were also upregulated in most of the Tx groups compared to the NTx groups. **c** mRNA expression levels of VEGF and VEGFR2 were increased in the 2- and 3-week Tx groups. **d** Immunofluorescence showed high expression of VEGFR2 in blood vessel epithelial cells in the ovaries. V, blood vessel. Scale bar: 25 μm. *vs. the normal group (*P* < 0.05). # vs. the NTx group (*P* < 0.05). Nor normal group, NTx non-transplantation group, Tx transplantation group.

In our previous reports, PD-MSCs were shown to secrete several types of proangiogenic cytokines, including VEGF [33]. Therefore, we analyzed the expression of VEGFR2 in ovarian tissues to investigate the mechanism underlying the effects of PD-MSCs on vascular structure. Immunohistochemical staining for VEGFR2 revealed strong expression in oocytes and granulosa cells of the normal and Tx groups compared to those of the NTx group at the site of ovarian follicle development (Fig. 4a). Upregulation of *Vegf* and *Vegfr2* was confirmed at the mRNA and protein levels in ovarian tissues (Fig. 4b, c). *Vegf* and *Vegfr2* mRNA expression levels were decreased in the NTx group compared to the normal group (*P < 0.05). However, the 2- and 3-week PD-MSC Tx groups showed increased expression of *Vegf* and *Vegfr2* compared to that in the respective NTx groups (*P < 0.05). *Vegfr1*, another member of the *Vegfr* family, and the proangiogenic factor endoglin also showed similar mRNA expression increases (Supplementary Fig. 2a, b). Western blot analysis also confirmed this upregulation. The protein levels of VEGF and VEGFR2 were increased in all groups except the 3-week Tx group (*P < 0.05). To confirm the localization of the high levels of these proteins, immunofluorescence was conducted. The data revealed that the blood vessels in the ovaries showed high expression of VEGFR2 (Fig. 4d).

**Fig. 5:**
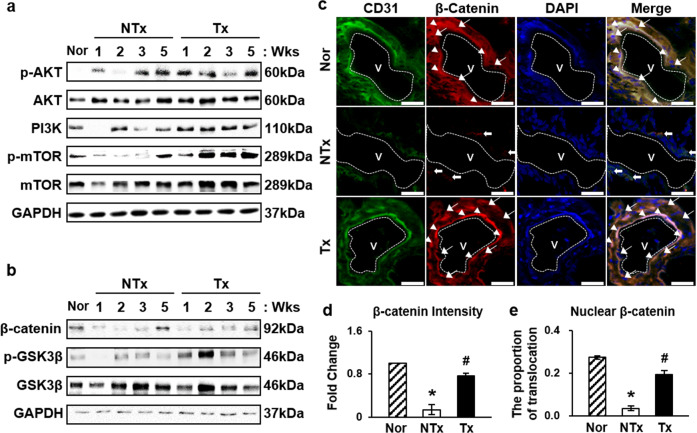
Activation of the PI3K/AKT/mTOR and GSK3β/β-catenin pathways. **a** Activation of the PI3K/AKT/mTOR pathway was induced by high expression of the phosphorylated forms of AKT, mTOR, and PI3K(p110α). **b** Protein levels of the phosphorylated form of GSK3β were increased. **c** Immunofluorescence showed high expression of CD31 (green), an epithelial cell marker, and β-catenin (red), and nuclear translocation of β-catenin merged with DAPI (blue). Scale bar: 25 μm. **d**, **e** Proportion of nuclear-translocated β-catenin was significantly higher in the Tx group than the NTx group. Arrowhead, β-catenin localized in the nucleus. Arrow, β-Catenin localized in the cytoplasm. *vs. the normal group (*P* < 0.05). #, vs. the NTx group (*P* < 0.05). Nor normal group, NTx non-transplantation group, Tx transplantation group.

The activation of the VEGF signaling pathway was evaluated by analyzing the expression levels of proteins related to the phosphatidyl inositol 3-kinase(PI3K)/AKT/mTOR and glycogen synthase kinase-3β(GSK3β)/β-catenin pathways. The PI3K/AKT/mTOR signaling pathway regulates many various biological processes, such as angiogenesis, and phosphorylation of AKT is known to be an indicator of PI3K/AKT/mTOR signaling pathway activation [34]. High levels of the active form of AKT were observed in the Tx groups compared to the NTx groups (Fig. 5a, Supplementary Fig. 3a, b). In addition, immunofluorescence verified the nuclear translocation of the nonphosphorylated form of β-catenin, which, once activated, acts as a transcription factor in the nucleus (Fig. 5c–e). These data indicate that engrafted PD-MSCs triggered the PI3K/AKT/mTOR signaling pathway and β-catenin activation in blood vessels in ovarian tissues.

### Increase in VEGF expression by PD-MSCs enhanced follicular development by upregulating VEGF signaling pathway-related proteins in injured ovarian tissues

**Fig. 6:**
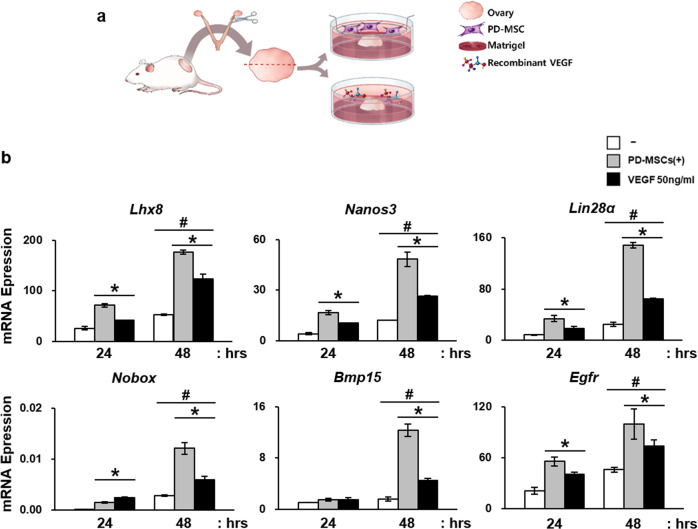
Enhancement of folliculogenic factors after cocultivation with PD-MSCs or treatment with recombinant VEGF ex vivo. **a** The scheme of the ex vivo experiment. Ovaries were harvested from a wild-type rat, cut in half, and treated with either PD-MSCs or recombinant VEGF. **b** The mRNA expression levels of folliculogenic factors were upregulated after cocultivation with PD-MSCs or treatment with recombinant VEGF (50 ng/ml). *vs. no treatment (*P* < 0.05). #, vs. the 24-h group (*P* < 0.05). $, vs. the PD-MSC-cocultivated group.

**Fig. 7:**
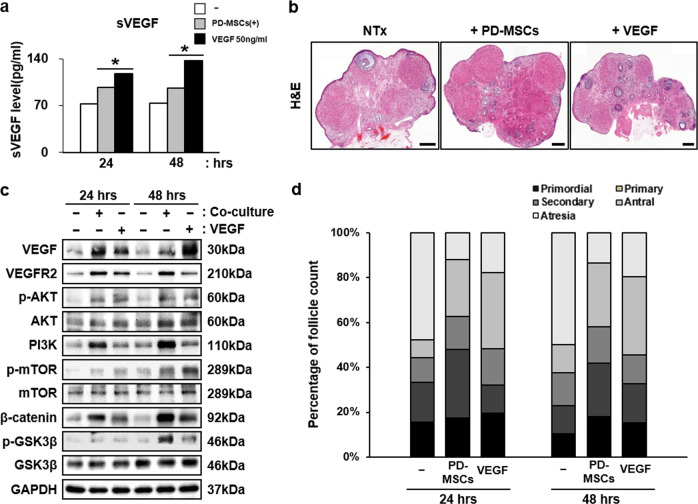
Activation of VEGF signaling pathways and increase in the number of mature follicles. **a** Enhanced expression of soluble VEGF in the media after cocultivation or VEGF treatment. **b** H&E-stained ovarian tissues. **c** The PI3K/AKT/mTOR and GSK3β/β-catenin pathways were highly activated after cocultivation or VEGF treatment. Scale bar (H&E): 500 μm. Scale bar (VEGFR2): 100 μm. **d** The number of mature follicle was increased in the PD-MSC-cocultivated group and VEGF-treated group.

Ex vivo cocultivation of PD-MSCs and rat ovaries was conducted to verify the results observed in vivo in an isolated experimental scheme (Fig. 6a). Using this experimental scheme, the paracrine interaction between PD-MSCs and ovaries was examined. The dose of VEGF recombinant was determined based on previously reports and preliminary experiments conducted by the authors to select the experimental dose using 3 different doses: e.g., 1 ng/ml, 50 ng/ml, and 100 ng/ml (Supplementary Fig. 4a) [35, 36]. As a results, the mRNA expression of *Lhx8, Nanos3, Lin28α, Nobox, Bmp15*, and *Egfr* was significantly increased in ovarian tissues cocultivated with PD-MSC compared to that of the group that was not cocultivated (Fig. 6b). Notably, treatment with recombinant hVEGF also induced upregulation of folliculogenic factors, but not as robustly as PD-MSCs. Moreover, the expression levels were increased in the 48-h groups compared to the 24-h group, implying that the effects of PD-MSCs intensified as the cocultivation time increased. The soluble form of VEGF was also detected at higher levels in the cocultivation and VEGF-treated groups compared to the NTx group (*P < 0.05) (Fig. 7a). Ovaries from each group were sectioned and stained with H&E for follicular analysis and lysed for western blot analysis (Fig. 7b, c). The protein expression levels of VEGF and VEGFR2 were also significantly upregulated, as observed in cocultured ovarian tissue samples (Fig. 7c). The associated signaling pathways were also analyzed to confirm the activation of VEGF signaling by examining the protein expression levels of PI3K, phospho-AKT, AKT, phospho-mTOR, and mTOR in the PI3K/AKT/mTOR pathway and β-catenin, phospho-β-catenin, and phospho-GSK3β in the GSK3β/β-catenin pathway (Fig. 7c, Supplementary Fig. 5a, b). Phospho-AKT, PI3K and phospho-mTOR were all upregulated in both the 24- and 48-h groups, implying that the PI3K/AKT/mTOR pathway was activated. β-catenin and phospho-GSK3β were also confirmed to be expressed at a high level, verifying the activation of the β-catenin pathway. Interestingly, compared to recombinant hVEGF treatment, PD-MSC cocultivation induced greater activation of the PI3K/AKT/mTOR and GSK3β/β-catenin pathways and higher VEGFR2 expression. In addition, the follicle count was also increased in the cocultivation group compared to the NTx group (*P < 0.05) (Fig. 7d). The numbers of total and antral follicle were increased in both the PD-MSC-cocultivated group and VEGF-treated group, but the cocultivation group showed a more dramatic increase than the VEGF treatment group. However, a time-dependent effect was not observed in this ex vivo experiment: there were no significant differences between the 24- and 48-h treatment results. These data indicate that high levels of VEGF and VEGFR2 expression by PD-MSCs promoted folliculogenesis in the ovary through activation of the VEGF signaling pathway.

## Discussion

Stem cell therapy using MSCs has shown great potential in regenerative medicine. Various MSCs have been utilized to treat ovarian dysfunction, such as POI and PCOS [37, 38]. However, a clear understanding of the mechanism is essential to maximize the efficiency of the outcome of this therapy. In this study, we demonstrated the proangiogenic effects of PD-MSCs on ovarian function and the close relationship between the ovarian vasculature and ovarian function in an OVX model and an ex vivo experimental scheme.

Among the many mechanisms underlying the therapeutic effects of MSCs, recent studies have focused on the paracrine mechanism of the proangiogenic effects of MSCs, which involves the secretion of bioactive molecules [39–42]. Umbilical cord MSCs have been reported to secrete several angiogenic factors, such as EGF and VEGF, which contribute to the proangiogenic effect of MSCs [39]. VEGF acts as a strong proangiogenic factor that significantly affects vascularization in the ovary, and this vascular structure is intimately correlated with folliculogenesis. Functional studies have revealed the role of VEGF in follicular angiogenesis by inactivating VEGF and observing the result of follicular development inhibition [43, 44]. In addition, placental chorionic villus MSCs are able to restore ischemic hind limbs by secreting angiogenic factors, VEGF and hepatocyte growth factor after Tx [40]. Furthermore, clinical studies have suggested that MSCs release proangiogenic factors to promote angiogenesis and increase local perfusion [41]. It has also been reported that VEGF is involved in follicle activation and blood vessel extension during follicular development in response to gonadotropic stimuli [45]. During the growth phase, follicles undergo a significant increase in volume. The rapidly growing cells consume a large amount of oxygen, which induces local hypoxia. This hypoxic condition induces the activation of hypoxia-inducible factor-1α, a transcription factor for many proangiogenic factors, such as VEGF, ANGPTs, platelet-derived growth factor, and basic fibroblast growth factor [46, 47]. The intraovarian VEGF/VEGFR2 signaling pathway is critical for gonadotropin-dependent follicular angiogenesis and development [48]. Using the CLARITY technique, Feng et al. and colleagues reported that ovarian tissue remodeling during folliculogenesis involves a close relationship between the ovarian vasculature and follicular development, revealing that more widespread vasculature within the ovary enhances folliculogenesis [49]. In addition, it has been reported that treatment with the proangiogenic isoform VEGFA164 induces denser vascularization and an increase in the number of follicles in the ovary [50].

Our study confirmed that PD-MSCs induced the upregulation of VEGF and VEGFR2 and the activation of their signaling pathways in injured ovarian tissues. In turn, they promoted vascular remodeling in the ovary, as assessed by histological analysis and blood vessels measurements taken with 3DHistech. Compared to the normal group, the OVX models showed enlargements of the blood vessel luminal area and wall thickness; however, the vessel wall thickness and luminal area were dramatically reduced after PD-MSC Tx than those without Tx, and the overall shape of the blood vessels were more circular structure, as in the normal group (Fig. 3a). Generally, the vascular structure, including the luminal area and wall thickness, is related to its function as a blood vessel to provide regulated blood flow, and OVX rats show dysregulated vascular function after expansion and thickening of the blood vessels in the heart [15, 51]. Therefore, our results may indicate that proper formation and structure of ovarian blood vessels are disrupted in this animal model after ovariectomy. However, after Tx, vascular structure was restored to a certain extent and allowed more regulated blood vessel function within the ovary. Nevertheless, the overall density of the vasculature in the ovary and the correlations between the structure and location of blood vessels and follicular growth should be addressed in future studies to determine the functional effects of the changes in the ovarian vasculature induced by PD-MSC Tx.

Using an ex vivo experimental scheme, we demonstrated the angiogenic and folliculogenic effects of PD-MSCs on the ovary and of treatment with recombinant human VEGF to verify the effect of VEGF alone on the ovary (Fig. 6a). As shown in Fig. 7, cocultivation of ovaries and PD-MSCs significantly upregulated folliculogenic factors and activated VEGF signaling pathways (Fig. 7c, d). Interestingly, treatment with recombinant VEGF alone also induced upregulation of folliculogenic factors, but to a lesser degree than cocultivation. These results indicate that the promotion of folliculogenesis and angiogenesis occurred via VEGF but that PD-MSC cocultivation induced greater enhancement, as PD-MSCs secrete many cytokines that positively influence the process of angiogenesis and folliculogenesis, including VEGF.

Furthermore, our results revealed that the VEGF signaling pathway was activated via the PI3K/AKT/mTOR pathway along with stimulation of the GSK3β/β-catenin pathway. VEGF, by binding and activating VEGFR2, has been reported to activate many intracellular signaling pathways, and one of them is PI3K/AKT pathway [52]. PI3K/AKT signals inactivation of GSK3β by phosphorylating GSK3β [53]. GSK3β degradates β-catenin through phosphorylation, so by inactivating GSK3β the active form of β-catenin may translocate from cytoplasm to the nucleus and actively perform as transcription factor to promote the expression of genes involved in cell proliferation [54–56]. Our data are similar to a previous report showing that MSCs derived from the human umbilical cord have a restorative effect on ovarian function in a mouse model of POI via activation of the PI3K/AKT signaling pathway and promotion of VEGF expression and thereby enhance angiogenic activity in the damaged ovary [57]. In addition, it has been suggested that bone marrow MSC Tx can ameliorate angiogenesis and reduce renal damage in rats by activating the PI3K/AKT signaling pathway and increasing serum VEGF levels [58]. These data support our conclusion that PI3K/AKT signaling triggered by PD-MSCs promotes angiogenesis through the nuclear translocation of β-catenin of angiogenesis-associated *Wnt* signaling (Fig. 5).

**Fig. 8:**
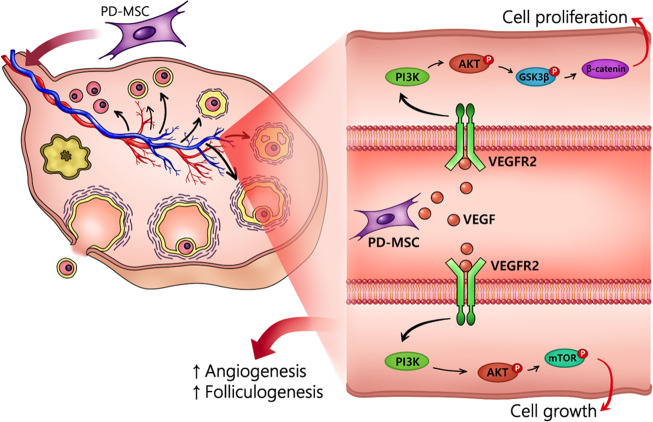
Graphical abstract of the effect of PD-MSCs on folliculogenesis. Secreted VEGF from PD-MSCs initiates the activation of endogenous VEGF signaling through the PI3K/AKT and GSK3β/β-catenin pathways, leading to vascular remodeling of ovarian blood vessels and, eventually, enhanced folliculogenesis.

In summary, our study suggests that transplanted PD-MSCs promoted increased secretion of VEGF, which, in turn, induced remodeling of the vascular structure in the OVX model and ultimately enhanced follicular development (Fig. 8). These results could be fundamental for understanding the therapeutic effects and mechanism of stem cell therapy with PD-MSCs and provide a theoretical foundation for their application for obstetrical and gynecological diseases, including infertility and menopause.

## Supplementary information

Supplementary Figures

Supplementary Table

APC payment form
