# Supplementary material for: Vascular remodeling by placenta-derived mesenchymal stem cells restores ovarian function in ovariectomized rat model via the VEGF pathway
Source: Lab Invest. 2020 Dec 10;101(3):304–17. doi: 10.1038/s41374-020-00513-1 (PMC7892345; doi:10.1038/s41374-020-00513-1)
Supplement: Supplementary file 1 — Supplementary Figures [file 41374_2020_513_MOESM1_ESM.pdf]

Supplementary Figure 1

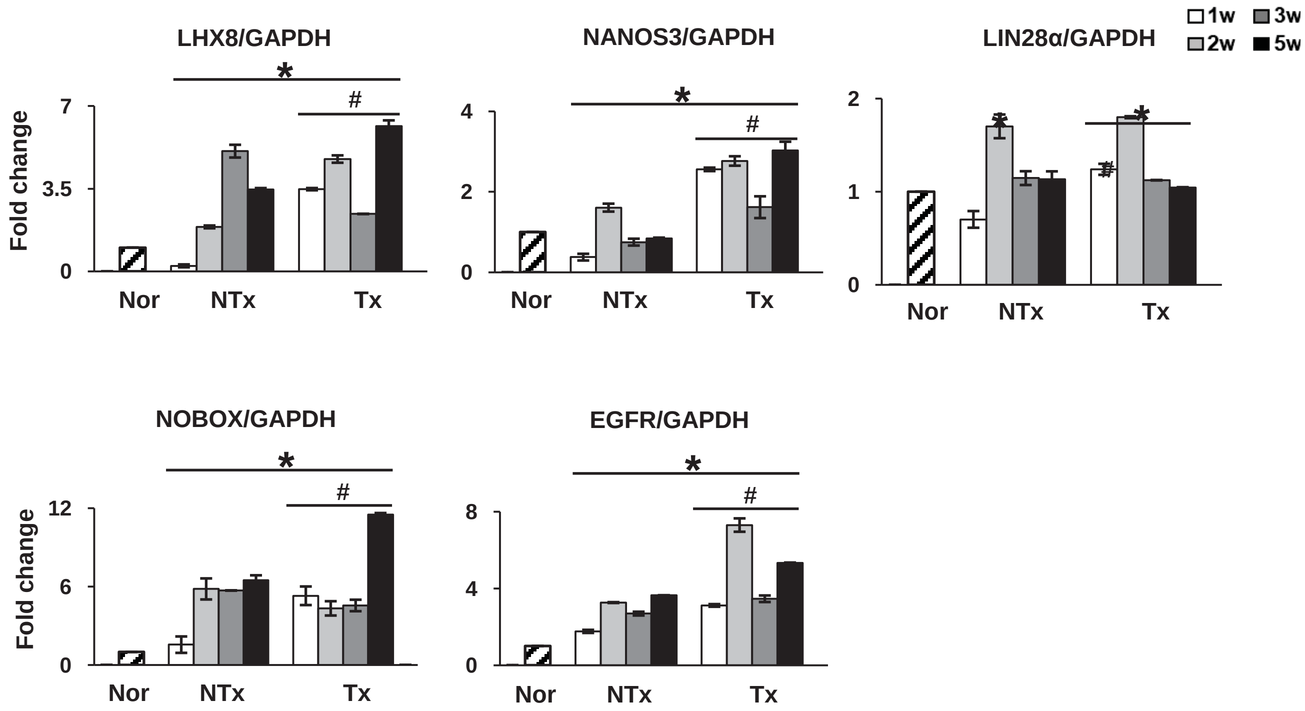

Supplementary Fig. 1 Graph of the expression of folliculogenesis factors, as determined by western blot analysis. Western blot analysis showed an overall increase in expression levels in the Tx group compared to the NTx group. * vs. the normal group (P<0.05). # vs. the NTx group (P<0.05). Nor, normal group. NTx, non-transplantation group. Tx, transplantation group. 1w, week 1. 2w, week 2. 3w, week 3. 5w, week 5.

Supplementary Figure 2

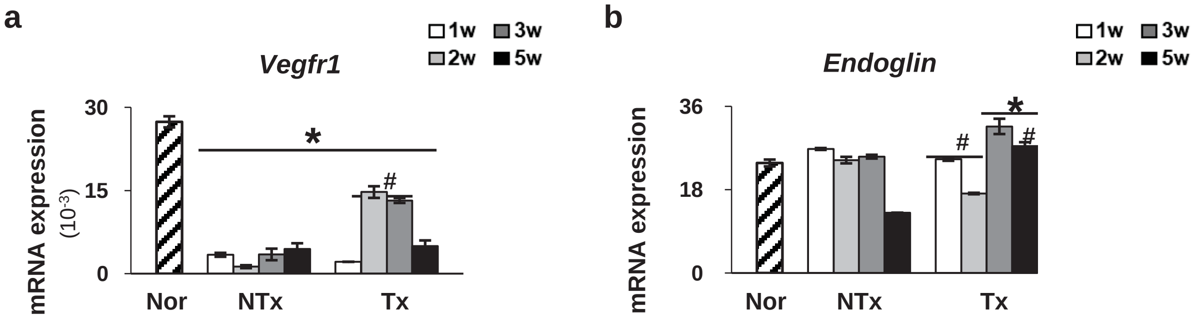

Supplementary Fig. 2 mRNA expression levels of *Vegfr1* and *Endoglin*. a, b The mRNA expression levels of *Vegfr1*, another member of the *Vegfr* family, and *Endoglin*, a proangiogenic factor, were increased in all groups. * vs. the normal group ($P<0.05$). # vs. the NTx group ($P<0.05$). Nor, normal group. NTx, non-transplantation group. Tx, transplantation group. 1w, week 1. 2w, week 2. 3w, week 3. 5w, week 5.

Supplementary Figure 3

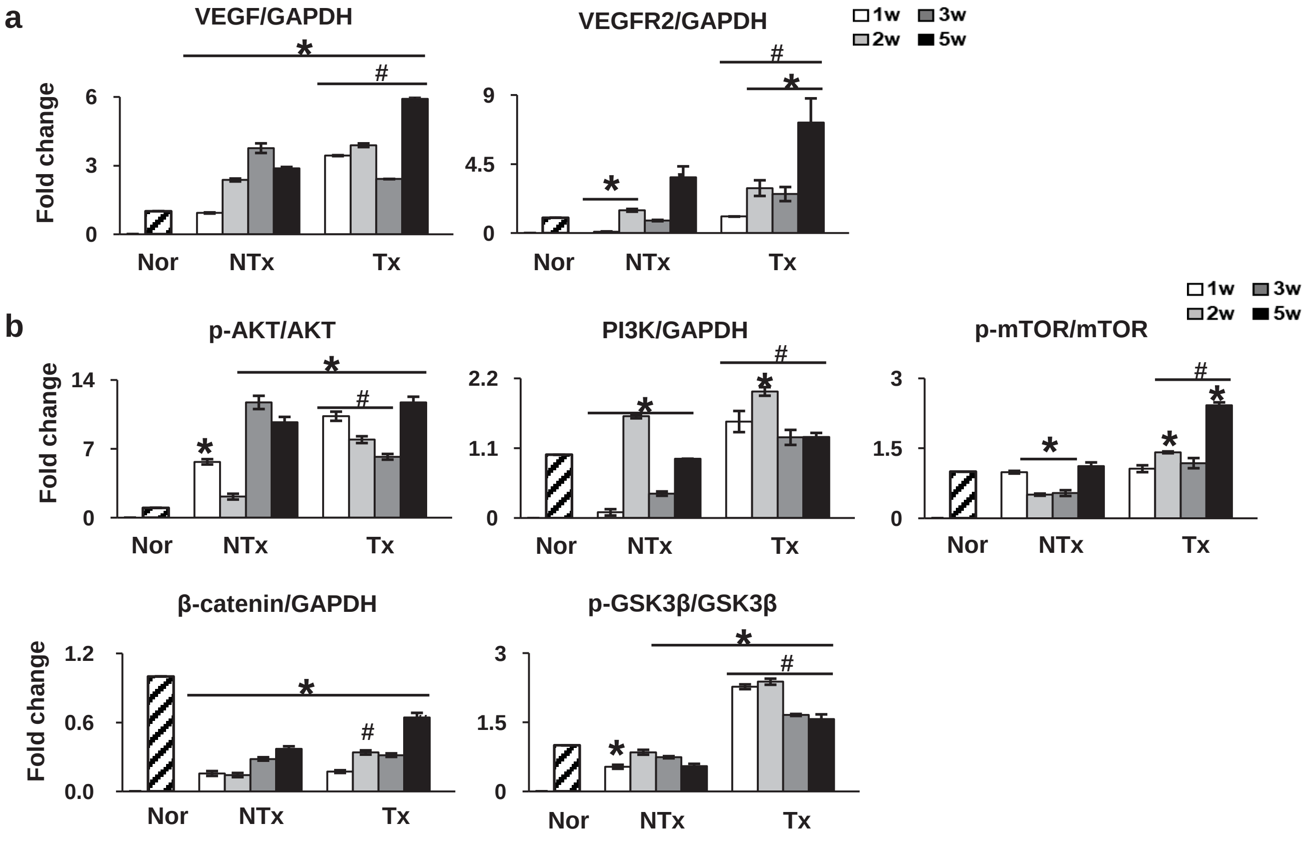

Supplementary Fig. 3 Graph of the expression of VEGF signaling pathway-related proteins in vivo, as determined by western blot analysis . a, b Western blot analysis showed an overall increase in expression levels in the Tx group compared to the NTx group. * vs. the normal group (P<0.05). # vs. the NTx group (P<0.05). Nor, normal group. NTx, non-transplantation group. Tx, transplantation group. 1w, week 1. 2w, week 2. 3w, week 3. 5w, week 5.

Supplementary Figure 4

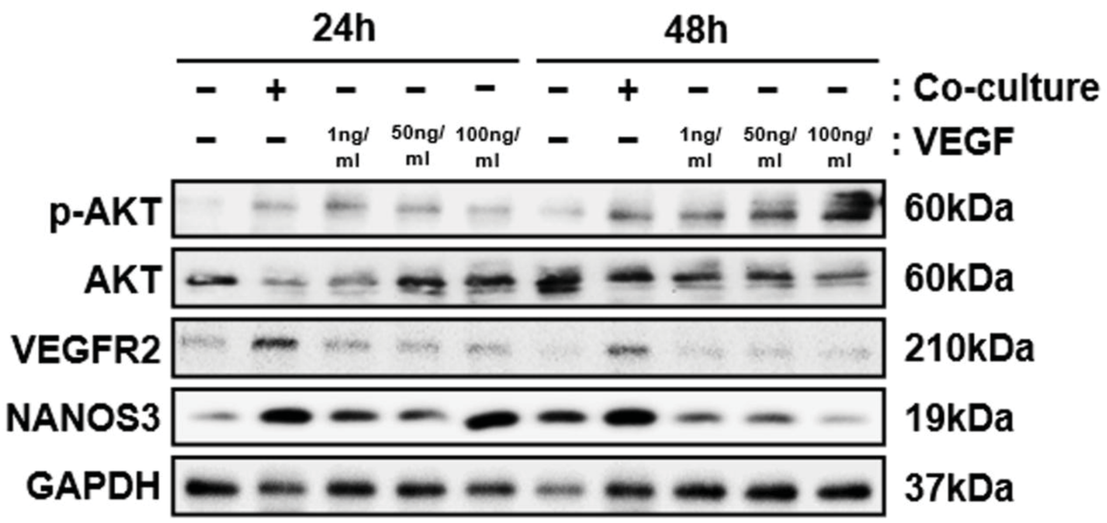

Supplementary Fig. 4 Preliminary experimental result of target gene expressions after PD-MSC co-cultivation or VEGF treatment according to dose dependent. Western blot analysis showed difference of target gene expression levels according to PD-MSC co-cultivation or dose dependent of VEGF recombinant.

Supplementary Figure 5

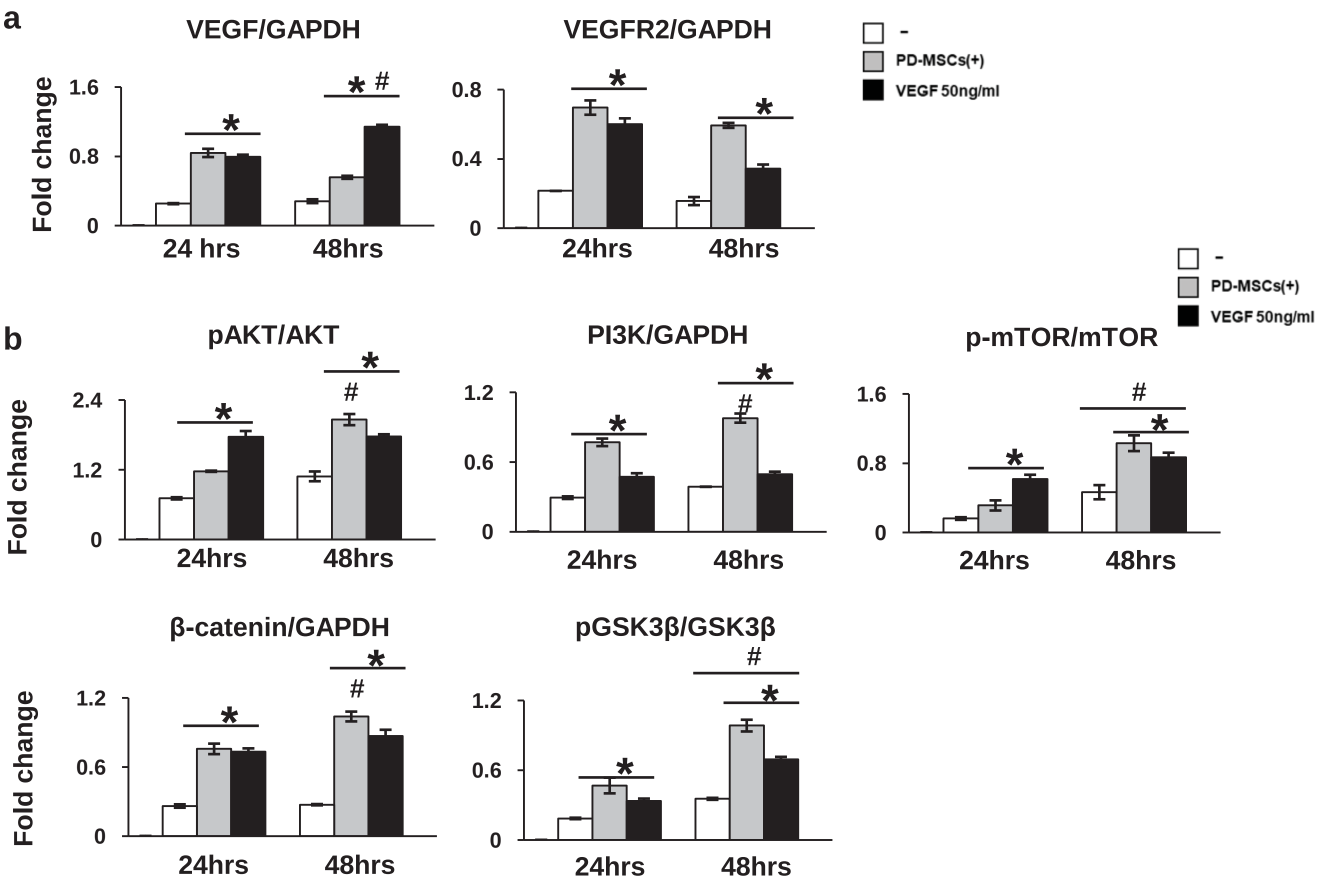

Supplementary Fig. 4 Graph of the expression of VEGF signaling pathway-related proteins ex vivo, as determined by western blot analysis. a, b Western blot analysis showed an overall increase in expression levels in the cocultivation group and/or VEGF-treated group compared to the NTx groups. The cocultivation group showed a higher increase than the VEGF-treated group in most cases. * vs. non-transplantation group (P<0.05). # vs. 24-hour group (P<0.05).
