# Supplementary material for: Vascular remodeling by placenta-derived mesenchymal stem cells restores ovarian function in ovariectomized rat model via the VEGF pathway
Source: Lab Invest. 2020 Dec 10;101(3):304–17. doi: 10.1038/s41374-020-00513-1 (PMC7892345; doi:10.1038/s41374-020-00513-1)
Supplement: Supplementary file 2 — Supplementary Table [file 41374_2020_513_MOESM2_ESM.doc]

**Supplementary Table 1. Comparison of follicle counts after transplantation in vivo**

|  | | Primordial | | Primary | | Secondary | | Antral | | Atresia | | Total | |
| --- | --- | --- | --- | --- | --- | --- | --- | --- | --- | --- | --- | --- | --- |
|  |  | Avg. | S.D. | Avg. | S.D. | Avg. | S.D.. | Avg. | S.D. | Avg. | S.D. | Avg. | S.D. |
| Nor | | 8.5 | 1.73 | 5.75 | 2.36 | 8.25 | 1.26 | 11.5 | 2.65 | 16.5 | 0.96 | 50.75 | 3.30 |
| NTx | 1w | 5 * | 1.41 | 7 | 1.41 | 8 | 0.00 | 4.5 * | 0.71 | 34.5 * | 2.12 | 59 | 4.24 |
|  | 2w | 0 * | 0.00 | 1.5 * | 0.71 | 8.5 | 2.12 | 9 | 1.41 | 17 | 2.83 | 36 * | 0.00 |
|  | 3w | 2 * | 1.41 | 6 | 2.83 | 8.5 | 0.71 | 5.5 * | 0.71 | 29 * | 1.41 | 51 | 1.41 |
|  | 5w | 1 * | 0.00 | 2.5 | 2.12 | 6.5 * | 0.71 | 5 * | 1.41 | 37.5 * | 2.12 | 52.5 | 3.54 |
| Tx | 1w | 10 | 0.00 | 8.5 | 0.71 | 13.5 | 2.12 | 15.5 ^#^ | 2.12 | 7.5 * ^#^ | 0.71 | 55 * | 1.41 |
|  | 2w | 9.75 ^#^ | 0.96 | 11.25 * ^#^ | 1.71 | 11 * | 1.63 | 11.75 | 2.63 | 12.5 * | 1.29 | 56.25 * ^#^ | 4.27 |
|  | 3w | 5.5 * ^#^ | 0.71 | 4 | 1.41 | 6.5 | 2.12 | 12 ^#^ | 1.41 | 17 ^#^ | 2.83 | 45 | 1.41 |
|  | 5w | 8.5 ^#^ | 0.71 | 9.5 * ^#^ | 0.71 | 7 | 1.41 | 10.5 | 3.54 | 16 ^#^ | 2.83 | 51.5 | 2.12 |

*, vs. Nor (P<0.05). #, vs. NTx (P<0.05). Nor, normal group. －, non-treatment group. PD-MSCs, PD-MSC-treatment group. 1w, week 1. 2w, week 2. 3w, week 3. 5w, week 5.Avg, average. S.D, the standard deviation.

Supplementary Table 2. Comparison of follicle counts after transplantation ex vivo

|  | | Primordial | | Primary | | Secondary | | Antral | | Atresia | | Total | |
| --- | --- | --- | --- | --- | --- | --- | --- | --- | --- | --- | --- | --- | --- |
|  |  | Avg. | S.D. | Avg. | S.D. | Avg. | S.D.. | Avg. | S.D. | Avg. | S.D. | Avg. | S.D. |
| 24h | − | 3 | 0.96 | 3.67 | 2.83 | 1.33 | 0.96 | 2 | 0.58 | 9.67 | 2.45 | 9.33 | 1.15 |
|  | PD-MSCs | 6.5 | 2.12 | 11.5 | 0.71 | 5.5 | 0.71 | 9.5 | 0.71 | 4.5 | 0.71 | 33 | 2.83 |
|  | VEGF | 5.5 | 0.71 | 3.5 | 0.71 | 4.5 | 3.54 | 9.5 | 3.54 | 5 | 1.41 | 23 | 7.07 |
| 48h | − | 2.5 | 2.12 | 3 | 1.41 | 4 | 1.41 | 3.5 | 0.71 | 11.5 | 2.12 | 12.5 | 2.83 |
|  | PD-MSCs | 6 | 1.41 | 8 | 1.41 | 5.5 | 0.71 | 9.5 | 2.12 | 4.5 | 0.71 | 29 | 1.41 |
|  | VEGF | 3.5 | 0.71 | 4 | 1.41 | 3 | 0.00 | 8 | 1.41 | 4.5 | 0.71 | 18.5 | 0.71 |

*, vs. − group (P<0.05). #, vs. 24-hour group (P<0.05).－, non-treatment. PD-MSCs, with co-cultivation of PD-MSC. VEGF, VEGF treatment (50ng/ml). Avg, average. S.D, the standard deviation.
