# Supplementary material for: Vascular remodeling by placenta-derived mesenchymal stem cells restores ovarian function in ovariectomized rat model via the VEGF pathway
Source: Lab Invest. 2020 Dec 10;101(3):304–17. doi: 10.1038/s41374-020-00513-1 (PMC7892345; doi:10.1038/s41374-020-00513-1)
Supplement: Supplementary file 3 — APC payment form [file 41374_2020_513_MOESM3_ESM.pdf]

Laboratory Investigation

SPRINGER NATURE

OPEN ACCESS ARTICLE PROCESSING CHARGE (APC) PAYMENT FORM

If you wish to take the open access option for your article, you must complete this payment form in addition to the Open Access License to Publish (LtP) form.
This will allow your manuscript (as both full-text HTML and PDF) to be made open access immediately upon publication, permanently available free of charge to all readers.

Completion of this form constitutes consent for the indicated article to be published on an open access basis in accordance with the Open Access LtP form and confirms you agree to the charges detailed below. A PDF invoice will be sent to the email address you supply below within 72 hours. The invoice will include instructions for payment, allowing you to pay by credit card, cheque or bank transfer. Credit terms are 30 days from date of invoice. Failure to pay your invoice within the stated credit term will result in the open access status of the article being rescinded, with the article being published and placed behind the paywall.
You may also be subject to restrictions on your ability to publish with Springer Nature or this title in the future, involvement of a third party debt collection agency and legal proceedings.

You may be identified as eligible under a transformative agreement. If you are, we will ask your institution if they agree to cover the APC under their agreement with Springer Nature. If your institution approves, no APC invoice will be raised using the details below. If they decline, you will be invoiced and responsible for arranging payment of the APC. If you would prefer to publish behind a paywall in case of rejection by your institution, please tick this box: ☐

It is mandatory to send this completed form and the Open Access LtP form to the return address (details below) to ensure your paper is published open access. If you do not wish your article to be made open access it is not necessary to complete this form.

The APC will be charged at the following rate:

Please select the appropriate option below:			EUR (€)	USD ($)	GBP (£)
✔	CC BY*: Creative Commons - Attribution	All article types	3060	3860	2560

The billing entity you will be invoiced by and the currency you will be charged in are solely determined by the billing country provided and cannot be changed to accommodate fluctuating exchange rates. VAT or local taxes will be added where applicable (in some cases, local taxes must be paid in your country and will not be added to your invoice): for details please see our support portal. For most Springer Nature-owned titles, page and colour charges are waived for open access articles, and no other publication charges apply in addition to the APC. Some titles owned by our society partners may charge page and colour charges in addition to the APC. Please check individual journal instructions for authors for further details.

* The Creative Commons Attribution (CC BY) License is preferred by many research funding bodies. We support use of this license as it is recommended for maximum dissemination and use of open access materials. Other Creative Commons licenses might be available - Please contact ORSupport@springernature.com if you would like to discuss an alternative Creative Commons license, quoting your manuscript number.

SUBMISSION DETAILS (COMPLETE ELECTRONICALLY)

Title of the article:
Vascular remodeling by placenta-derived mesenchymal stem cells restores ovaria

Manuscript number:	Corresponding author's name:
20-0307-ARR	Gi Jin Kim
Corresponding author's email address:	Corresponding author's primary affiliation:
gjkim@cha.ac.kr	CHA University

BILLING DETAILS (COMPLETE ELECTRONICALLY)

Contact name:	Email address:
Gi Jin Kim	gjkim@cha.ac.kr
Telephone number:	PO Number (optional):
82-31-881-3687	
Organisation:	Department:
CHA University	Department of Biomedical Science
Billing address:	
335 Pangyo-Ro, Bundang-Gu	
Postcode/ZIP:	City/Town:
13488	Seongnam/Gyeonggi-Do
Billing country:	VAT/GST/ABN/Tax Number (optional):
Republic of Korea	

Please return your completed APC and License to Publish Forms to:
Dr Catherine Ketcham, Managing Editor - Laboratory Investigation
Email: labinvest@comcast.net
